# Association between *Staphylococcus aureus* nasal carriage and disease phenotype in patients affected by systemic lupus erythematosus

**DOI:** 10.1186/s13075-016-1079-x

**Published:** 2016-07-30

**Authors:** Fabrizio Conti, Fulvia Ceccarelli, Giancarlo Iaiani, Carlo Perricone, Alessandra Giordano, Luigino Amori, Francesca Miranda, Laura Massaro, Viviana Antonella Pacucci, Simona Truglia, Gabriella Girelli, Azis Fakeri, Gloria Taliani, Chiara Temperoni, Francesca Romana Spinelli, Cristiano Alessandri, Guido Valesini

**Affiliations:** 1 Lupus Clinic, Dipartimento di Medicina Interna e Specialità Medica, Sapienza Università di Roma, Viale del Policlinico 155, 00161 Rome, Italy; 2 DAI Malattie Infettive e Tropicali, Azienda Policlinico Umberto I, Rome, Italy; 3 Sanità Pubblica e Malattie Infettive, Sapienza Università di Roma, Rome, Italy; 4 UOC Immunoematologia e Medicina Trasfusionale, Azienda Policlinico Umberto I, Rome, Italy; 5 Dipartimento Medicina Clinica, Sapienza Università di Roma, Rome, Italy

**Keywords:** Systemic lupus erythematosus, Microbiome, *Staphylococcus aureus*

## Abstract

**Background:**

*Staphylococcus aureus* (SA) is a commensal bacterium representing one of the most important components of the skin microbiome, mostly isolated in the anterior nares. A higher rate of SA nasal colonization in patients affected by Wegener’s granulomatosis and rheumatoid arthritis compared with healthy subjects (HS) has been described. No studies focusing on systemic lupus erythematosus (SLE) are available. We aimed at analyzing the prevalence of SA nasal carriers in an SLE cohort and evaluating correlation between nasal colonization and clinical, laboratory and therapeutic features.

**Methods:**

We enrolled 84 patients with SLE (number of male/female patients 6/78; mean age 41.3 ± 12.2 years, mean disease duration 142.1 ± 103.8 months) and 154 HS blood donors. Patients with SLE underwent a physical examination and the clinical/laboratory data were collected. All the patients with SLE and the HS received a nasal swab for SA isolation and identification.

**Results:**

SA nasal colonization prevalence was 21.4 % in patients with SLE and 28.6 % in HS (*P* not significant). We analyzed patients with SLE according to the presence (n = 18, SA-positive SLE) or the absence (n = 66, SA-negative SLE) of nasal colonization. Renal involvement was significantly more frequent in SA-positive SLE (11.6 % vs 3.0 %; *P* = 0.0009). Moreover, the presence of anti-dsDNA, anti-Sm, anti-SSA, anti-SSB, anti-RNP antibodies was significantly higher in SA-positive SLE (*P* < 0.0001, *P* = 0.01, *P* = 0.008, *P* = 0.03, *P* = 0.03, respectively).

**Conclusion:**

SA colonization is a relatively frequent condition in patients with SLE, with a frequency similar to HS. The presence of SA seems associated with a peculiar SLE phenotype characterized by renal manifestations and autoantibody positivity, confirming the role of the microbiome in disease phenotype.

## Background

As widely demonstrated, genetic and environmental factors interplay in the development of autoimmune diseases, such as systemic lupus erythematosus (SLE) [1]. Several environmental factors have been implicated in the different pathological conditions, and great emphasis has been placed on the role of infection [2].

In recent years there has been growing interest in the possible role of the microbiome in the development and course of disease. Of note, the gut microbiome has been widely investigated in autoimmune diseases, such as type 1 diabetes, inflammatory bowel diseases, rheumatoid arthritis (RA), and spondyloarthropathies [3]. Conversely, few data are available on the skin microbiome and the relationship with autoimmune diseases. *Staphylococcus aureus* (SA) is a commensal microorganism and represents one of the most important components of the human skin microbiome [4]. SA is characterized by very heterogeneous pathogenic features, ranging from minor and self-limiting skin infections, such as impetigo, folliculitis, and furuncles, to invasive and life-threatening diseases, such as septic arthritis, osteomyelitis, meningitis, septicemia and staphylococcal toxic shock syndrome [5].

The anterior naris is the most frequent carriage site for SA, due to specific anatomical and biochemical characteristics facilitating the persistence of SA [6]. Data from the National Health and Nutrition Examination Survey 2001–2002 described a frequency up to 30 % of SA colonization in the general population in the USA [7]. A large cohort constituted by nine European countries described a frequency of SA carriage of 21.6 %, with lower values in the older population [5]. In the great majority of the cases, this colonization is intermittent and only in 20 % of cases is persistent [6].

Very few studies have evaluated the prevalence of SA nasal carriage in patients affected by autoimmune diseases and its association with the specific disease phenotype. In 1996 Tabarya and colleagues described a prevalence of SA carriers of 50 % among patients with RA from a cohort of 88 individuals, compared with 33 % identified in a healthy control population [8]. More recently, in 2005 Bassetti et al. did not identify any significant difference in SA carrier prevalence, between RA and a group of patients without, enrolled as controls (34.5 % versus 32.5 %). Moreover, concomitant treatment with tumor necrosis factor (TNF) antagonists and methotrexate appeared to be the only independent factor associated with carriage of nasal SA (OR 3.24) [9]. Conversely, a relationship between SA and granulomatosis with polyangiitis (GPA) has been identified, suggesting the role of this specific bacterium in disease development and relapse [10]. Moreover, the study conducted by Laudien and colleagues demonstrated a significantly higher rate of SA nasal carriage in patients with GPA compared to a cohort of patients with RA and staff members (72.0 %, 46 %, and 58 %, respectively). Notably, the risk of relapse was higher in patients with GPA who had evidence of nasal SA [11].

Starting from the lack of studies in patients with SLE, in the present analysis we aimed at assessing the prevalence of SA nasal carriers in a monocentric SLE cohort and evaluated the association between SA nasal colonization and disease phenotype.

## Methods

Over a 3-month period, we enrolled 84 consecutive patients with SLE who had been referred to the Lupus Clinic of the Rheumatology Unit, Sapienza University of Rome (Sapienza Lupus Cohort). The diagnosis was performed according to the revised 1997 American College of Rheumatology (ACR) criteria [12]. One hundred fifty-four healthy blood donors were enrolled as the control group. Both patients and control subjects provided written informed consent at the time of the visit.

At each visit, patients with SLE underwent a complete physical examination. The clinical and laboratory data were collected in a standardized, computerized, and electronically filled form, including demographics, past medical history with the date of diagnosis, comorbidities, and previous and concomitant treatments. Disease manifestation was recorded according to the ACR classification criteria [12].

### Laboratory evaluation

The study protocol included the determination of autoantibodies and the evaluation of C3 and C4 serum levels. Antinuclear antibodies (ANA) were determined by means of indirect immunofluorescence (IIF) on HEp-2 (titer ≥1:160 or ++ on a scale from + to ++++), anti-double-stranded DNA (dsDNA) with IIF on *Crithidia luciliae* (titer ≥1:10), ENA (including anti-Ro/SSA, anti-La/SSB, anti-Sm, and anti-RNP) analyzed by enzyme-linked immunosorbent assay (ELISA) considering titers above the cutoff of the reference laboratory, anti-cardiolipin (anti-CL) (IgG/IgM isotype) analyzed by ELISA, in serum or plasma, at medium or high titers (e.g., >40 GPL or MPL or above the 99th percentile), anti-*β*2 Glycoprotein-I (anti-*β*2GPI) (IgG/IgM isotype) analyzed by ELISA, in serum (above the 99th percentile), and lupus anticoagulant (LA), according to the guidelines of the International Society on Thrombosis and Hemostasis (scientific subcommittee on lupus anticoagulant/phospholipid-dependent antibodies) [13]. Finally, C3 and C4 serum levels were determined by means of radial immunodiffusion.

### Disease activity and chronic damage

Disease activity was assessed by using the SLE Disease Activity Index 2000 (SLEDAI-2 k) and the European Consensus Lupus Activity Measurement (ECLAM) [14, 15]. For all patients with SLE, the occurrence of flare and the presence of a persistently active disease in the 12 months prior to and the 12 months following the visit were registered. Specifically, a flare was defined as an increase in SLEDAI-2 K score ≥4 from the previous visit, with a minimum interval of 2 months between visits and persistently active disease as an SLEDAI-2 K score ≥4, excluding serology alone, on two or more consecutive visits [16]. Finally, the SLICC Damage Index (SDI) was applied to assess chronic damage [17].

### Nasal swabbing

Both anterior nares were swabbed following a standard operational procedure. BBL™ Culture Swab™ Collection and Transport System (Made by Copan for Becton, Dickinson, and Company, Sparks, USA) was used. Specifically, only one swab was used for both nares. The swab should be inserted in the nasal vestibule, introducing only the cotton part of the swab. The operator should rotate the swab while circulating in the nasal vestibule for approximately 5 seconds. This procedure had to be repeated in both nares [18]. Nasal swabbing was performed at baseline and after specific treatment in subjects positive for SA.

### Microbiological evaluation

The total bacteria and colony forming units (CFU) of each single bacterial strain, were analyzed for every single swab. Bacteria were quantified by the standard plate count method, in which the bacteria are grown in a nutrient culture and developing colonies counted. This method entails diluting a sample with a specific buffer diluent until the bacteria are sufficiently diluted to count accurately, with the formation of colonies. In particular, the CFU was used to estimate the number of viable bacteria in the sample. Specifically, swabs were cultured on Columbia agar supplemented with 5 % sheep blood agar at 37 °C under the ambient atmosphere for 48 h. CFUs were then counted by macroscopic inspection. SA was distinguished from *Staphylococcus epidermidis* by hemolysis (b-hemolysis versus no hemolysis) and colony color (golden yellow versus white), if necessary by agglutination assay (Slidex Staph Plus, bioMérieux). Carrier state was defined as a condition characterized by identification of SA on the nasal swab analysis in individuals with no symptoms of skin or respiratory infection.

### Treatment of SA nasal carriage

According to the protocol, all SA-positive patients were treated by application of Mupirocin 2 % twice per day to the nares for 5 days; this treatment was repeated monthly for 12 months [19].

### Epidemiologic background information

Both patients with SLE and healthy controls were invited to fill a questionnaire in order to identify the presence of factors increasing the risk of becoming an SA carrier. In particular, the following information was registered:

1. Interaction with pets other than fish, specifying the type
2. Contact with other animals, specifying the number of times a week,
3. Hospitalization within 90 days (in the case of a positive answer, the patient/subject had to specify the reason and therapy)
4. Antibiotic treatment within 90 days (in the case of a positive answer, the patient/subject had to specify the reason and therapy)
5. Living with health care, veterinary, prison operators
6. Team sports practice
7. Previous SA infection requiring antibiotic treatment

### Statistical evaluation

We used version 13.0 of the SPSS statistical package. Normally distributed variables were summarized using the mean ± standard deviation (SD) and non-normally distributed variables by the median and range. Percentages were used when appropriate. The Mann-Whitney test was performed accordingly. Univariate comparisons between nominal variables were calculated using the chi-squared test or Fisher’s test where appropriate. Multivariate analysis was performed using binary logistic regression. In order to perform the multivariate analysis, we used a step-forward model including, progressively, those variables with *P* < 0.1 (as were those with a trend towards significant association) to produce a stronger model. Two-tailed *P* values were reported. *P* values <0.05 were considered significant. Based on the number of patients enrolled, and lacking data on the prevalence of SA nasal carriage in patients with SLE, we planned a study with 1.8 controls per case. The sample size was estimated by evaluating prior data in which the prevalence of SA carriers among patients affected with RA ranged between 34.6 and 46 % [8, 9], while in the larger cohort reported previously, the prevalence of SA carriers among the general population was 21.6 % [5]. Thus, if the true probability of exposure among cases was 40 %, we needed to study at least 74 case patients and 133 controls to be able to reject the null hypothesis that the exposure rates for cases and controls were equal with probability (power) 0.8, with a type I error probability (alpha) of 0.05.

## Results

We enrolled 84 Caucasian patients with SLE and 154 healthy controls (HC). In Table 1 demographic, clinical, laboratory and treatment data for patients with SLE enrolled in the present study are described: all data refer to the disease history. Healthy controls were 109 men and 45 women; mean age ± SD was 40.9 ± 9.7 years. There were 20 patients with SLE who had concomitant autoimmune diseases: 13 patients (65.0 %) with antiphospholipid syndrome and 7 (35.0 %) with Sjögren’s syndrome. No significant difference was found in mean age between patients with SLE and HC; conversely, male gender was significantly more frequent in HC (41.3 %) compared with patients with SLE (7.7 %; *P* < 0.001).

**Table 1:** Demographic, clinical, laboratory features and used treatments of patients with systemic lupus erythematosus (n = 84)

Demographic features	Value
Male/female, *n*	6/78
Mean age ± SD (years)	41.3 ± 12.2
Mean disease duration ± SD (months)	142.1 ± 103.8
Clinical manifestations, *n* (%)	
Joint involvement	57 (67.8)
Skin involvement	58 (69.0)
Serositis	18 (21.4)
Hematological manifestations	37 (44.0)
Neuropsychiatric involvement	10 (11.9)
Renal involvement	31 (36.9)
Laboratory manifestations, *n* (%)	
Antinuclear antibodies	84 (100.0)
Anti-DNA	69 (82.1)
Anti-Sm	13 (15.5)
Anti-SSA	24 (28.6)
Anti-SSB	10 (11.9)
Anti-RNP	10 (11.9)
Anti-cardiolipin IgG/IgM	30 (35.7)
Anti-β_2_Glicoprotein I IgG/IgM	8 (9.5)
Lupus anticoagulant	12 (14.3)
Low C3 levels	32 (38.1)
Low C4 levels	44 (52.4)
Treatments, *n* (%)	
Corticosteroids	60 (71.4)
Hydroxychloroquine	54 (64.3)
Cyclosporine A	19 (22.6)
Methotrexate	17 (20.2)
Cyclophosphamide	19 (22.6)
Mycophenolate mofetil	15 (17.8)
Azathioprine	18 (21.4)
ASA	25 (29.7)
Anticoagulant therapy	9 (10.7)

### Comparison between SLE cases and healthy controls

Eighteen patients with SLE were nasal SA carriers (21.4 %). This percentage was similar to that reported in HC, in which 44 subjects were SA-positive (28.6 %; *P* = 0.3). Relevantly, in all cases, the identified SA strains were methicillin-sensitive. No significant differences in gender or age were identified in SLE or HC SA carriers (Table 2). Moreover, in patients with SLE no association between disease duration and SA colonization was observed (Table 2). The evaluation of other factors associated with nasal colonization demonstrated a higher frequency of antibiotic treatment within 90 days from the assessment in SA-positive subjects (SLE 16.7 %, HC 10 %) compared with SA-negative subjects (SLE 12.2 %, HC 3.2 %; *P* not significant (NS)). The frequency of the risk factors associated with SA carriage is described in Table 3 for both SLE and HC.

**Table 2:** Epidemiological features in patients with systemic lupus erythematosus (SLE) and healthy control (HC) subjects according to nasal carriage status

	SLE (n = 84)	HC (n = 154)	*P*
Nasal SA carrier prevalence (%)	21.4	28.6	NS
Male/female, *n*			
SA-positive	1/17	32/12	
SA-negative	5/61	77/33	NS
Mean age ± SD (years)			
SA-positive	40.9 ± 11.8	38.7 ± 12.6	NS
SA-negative	41.3 ± 12.4	41.6 ± 8.9	
Mean disease duration ± SD (months)			
SA-positive	150.0 ± 107.1	-	NS
SA-negative	139.6 ± 103.6	-	

*SA Staphyloccocus aureus, NS* not significant

**Table 3:** Epidemiologic background information in patients with systemic lupus erythematosus (SLE) and healthy controls (HC)

	SLE	HC	*P*
Interaction with pets (%)			
SA-positive	16.7	20.0	NS
SA-negative	24.4	22.6	
Contacts with other animals (%)			
SA-positive	22.2	0	*P* < 0.0001 SLE SA-positive vs HC SA-positive
SA-negative	12.2	12.9	*P* = 0.0001 HC SA-negative vs HC SA-positive
Hospitalization within 90 days (%)			
SA-positive	0	0	NS
SA-negative	1.5	0	
Antibiotic treatment within 90 days (%)			
SA-positive	16.7	10.0	
SA-negative	12.2	3.2	*P* = 0.02 SLE SA-negative vs HC SA-negative
Team sports practice (%)			
SA-positive	5.5	30.0	*P* < 0.001 SLE SA-positive vs HC SA-positive
SA-negative	0	3.2	*P* < 0.001 HC SA-positive vs HC SA-negative
Previous SA infections (%)			
SA-positive	0	0	NS
SA-negative	3.0	0	

### Comparison between SA-positive (SA+) and SA-negative (SA-) patients with SLE

**Fig. 1:**
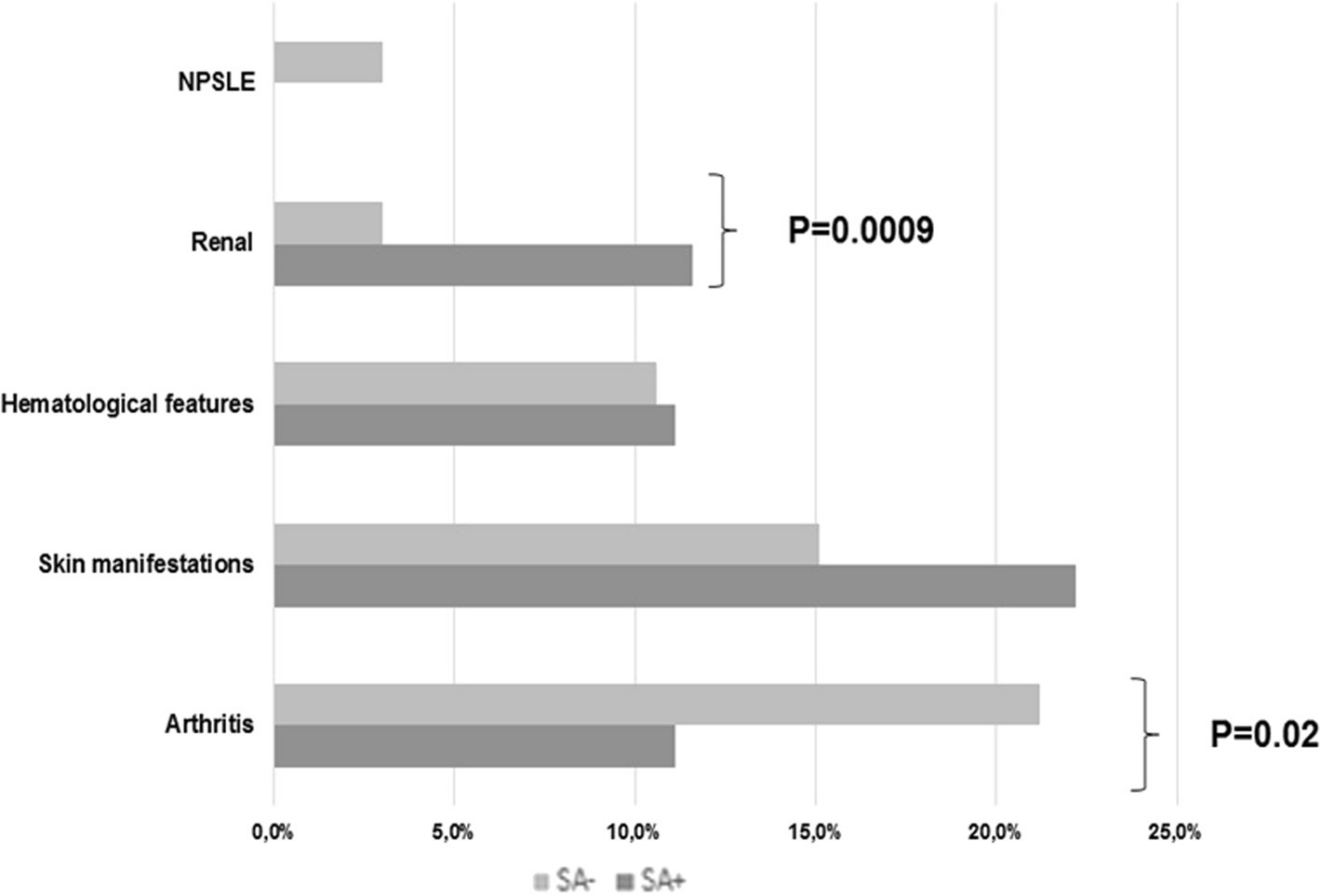
Clinical features in *Staphyoloccocus aureus*-positive (*SA*+) and SA-negative (*SA*-) patients with systemic lupus erythematosus at the time of enrollment. NPSLE: Neuropsychiatric SLE

**Fig. 2:**
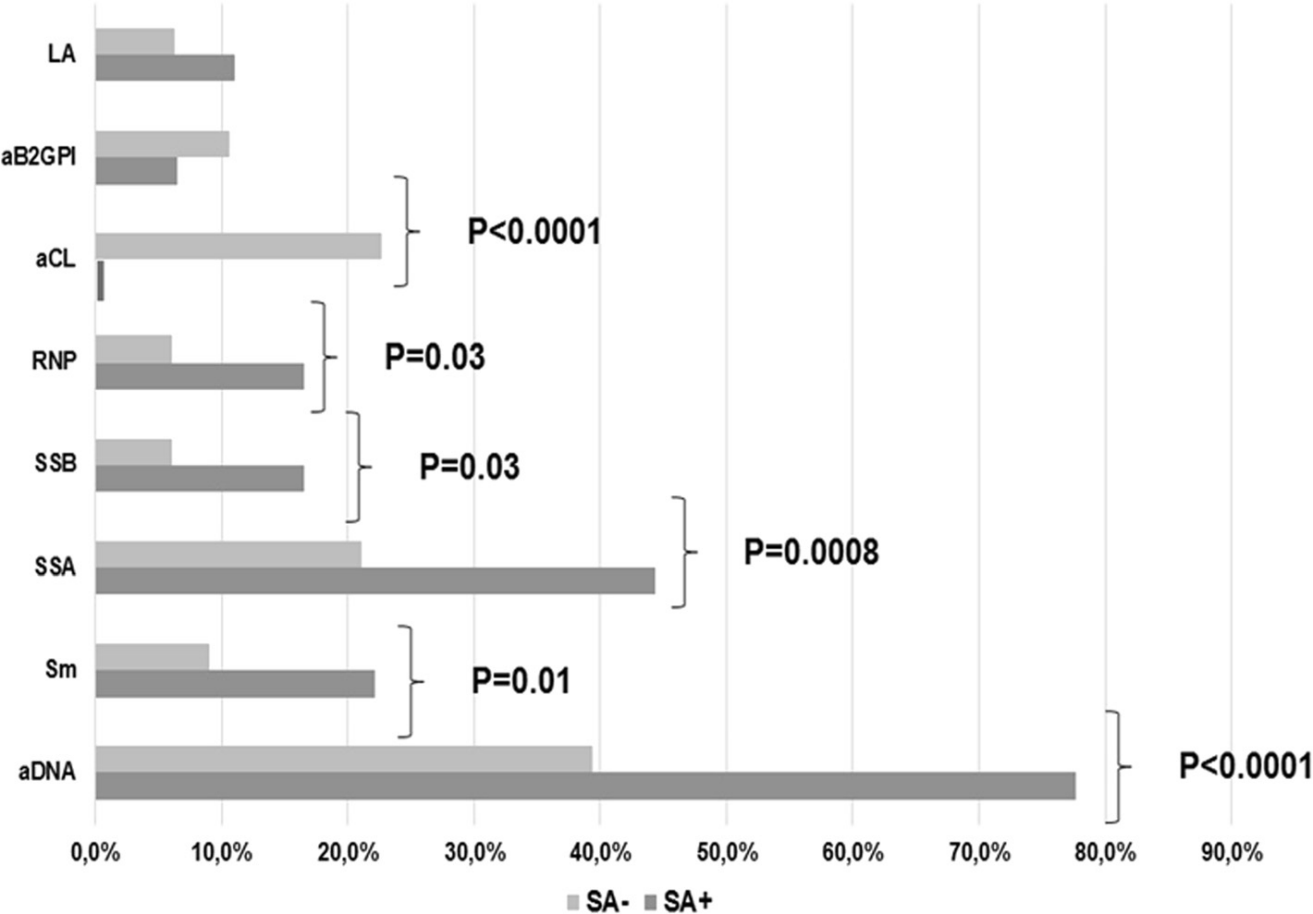
Laboratory features in *Staphyoloccocus aureus*-positive (*SA*+) and SA-negative (*SA*-) patients at the time of enrollment. LA: Lupus Anticoagulant; aB2GPI: anti-β Glycoprotein I, aCL: anti-cardiolipin

We then compared the clinical and laboratory features of SA+ and SA- patients with SLE. The frequencies of clinical manifestations in the two SLE groups at the time of the study entry are reported in Fig. 1. Renal involvement at the time of enrollment was significantly more frequent was in SA+ patients with SLE, compared with SA- patients (11.6 % vs 3.0 %; *P* = 0.0009). Similarly, although not statistically significant, a higher frequency of skin manifestations was observed in SA+ patients (22.1 %) compared with SA- patients (15.1 %; *P* = NS). Conversely, joint involvement was more frequent in SA- patients (21.2 % vs 11.1 %; *P* = 0.02). The evaluation of the autoantibody status in SA+ and SA- patients with SLE demonstrated a similar frequency of ANA (100 % versus 89.4 %; *P* = NS) at the time of the nasal swab. Interestingly, a significantly higher prevalence of anti-dsDNA, anti-Sm, anti-SSA, anti-SSB, and anti-RNP antibodies was identified in SA+ patients with SLE (77.7 %, 22.2 %, 44.4 %, 16.6 %, and 16.6 %, respectively) compared with SA- patients (39.4 %, *P* < 0.0001; 9.1 %, *P* = 0.01; 21.1 %, *P* = 0.0008; 6.1 %, *P* = 0.03; and 6.1 %, *P* = 0.03, respectively; Fig. 2). The results differed for anti-phospholipid antibodies: aCL positivity was observed only in SA- patients with SLE. Moreover, there were no significant differences between SA+ and SA- patients with SLE in the prevalence of LA and anti-β_2_GPI.

C3 and C4 levels were more frequently reduced in SA- patients with SLE (low C3 levels: 19 patients (29.7 %) SA- vs 3 patients SA+ (16.6 %); *P* = 0.01; low C4 levels: 21 patients (32.8 %) SA- vs 4 patients SA+ (22.2 %; *P* = 0.03). The evaluation of SLE treatment in the two groups of patients revealed significantly more glucocorticoid treatment in the SA+ SLE group (15 patients (83.3 %)) compared with the SA- group (43 patients (65.1 %); *P* = 0.01). There were no significant differences in the mean weekly prednisone (or equivalent) dosage (SA+ patients 40.2 ± 21.3 mg/week vs 49.1 ± 41.1 mg/week; *P* = NS). The multivariate analysis confirmed the association between SA carriage status and positivity for anti-dsDNA (*P* = 0.003).

We also evaluated disease activity and chronic damage at the time of study enrollment. We did not find significant differences between SA+ and SA- patients with SLE in SLEDAI-2 k values (3.1 ± 3.6 vs 2.5 ± 2.4; *P* = NS), ECLAM (1.0 ± 0.1 vs 1.0 ± 0.9, *P* = NS) and SDI (0.1 ± 0.3 vs 0.3 ± 0.7, *P* = NS). In order to evaluate the role of SA nasal colonization in disease activity modification, we evaluated the frequency of persistently active disease and flares in the 12 months preceding study entry. We identified a trend towards higher frequency of persistently active disease in SA+ patients with SLE (27.7 %) compared with SA- patients (17.2 %; *P* = NS). A similar prevalence of flares was identified in the two groups in the previous 12 months (SA+ patients 11.1 %, SA- patients 9.3 %; *P* = NS).

### Treatment and follow up of SA+ patients with SLE

All the SA+ patients with SLE were treated with Mupirocin 2 % twice per day to the nares for 5 days. A nasal swab was repeated 2 weeks after the first treatment, demonstrating the absence of SA colonization in all the cases re-evaluated. Moreover, no differences were found before and after treatment in the frequency of flares (two flares (11.1 %) in the two groups over the 12-month follow up) and persistently active disease (before eradication: 5 patients (27.7 %); after eradication: 4 patients (22.2 %)).

## Discussion

In the present study, for the first time we evaluated the prevalence of SA nasal colonization in a cohort of patients affected by SLE. Despite a similar frequency of SA being observed in patients and a healthy control group, the SA colonization in patients with SLE was associated with a specific disease phenotype, characterized by renal and skin involvement, and a higher prevalence of a broad spectrum of autoantibodies.

SLE is an autoimmune disease characterized by very heterogeneous autoantibody production and clinical manifestations [1]. Infectious agents seem to play an important role in disease pathogenesis due to their ability to activate B-cell-mediated and T-cell-mediated autoimmune responses leading to the production of autoantibodies [2, 20]. More recently, the interferon (IFN) signature was shown as an additional mechanism involved in the disease, confirming the possible role of infection [21]. Moreover, innate immunity through pathogen-associated molecular patterns and Toll-like receptors (TLRs) may also contribute to disease development [22].

The microbiome is a novel and intriguing concept that has been reported to be involved in the pathogenesis of several autoimmune diseases. Most studies focused on the gut microbiome; nonetheless, the skin microbiome could play a role in human autoimmune conditions. Changes in the skin microbiome seem to influence the disease course through the modulation of the cutaneous immune system. Moreover, each individual has a unique skin microbiome influenced by pH, salinity, sebum content of the topographical body region, and by intrinsic (e.g. genotype, age, and sex) and extrinsic individual-dependent factors (e.g., occupation, geographical location, smoking, sun exposure, and use of antibiotics or cosmetics) [4]. SA is one of the components of the skin microbiome that could potentially colonize all body surfaces including the gut and anterior nares [5, 6].

It seems to induce an inflammatory response by exposing staphylococcal superantigen, molecular mimicry, causing increased TLR signaling in leukocytes and inducing neutrophil extracellular traps [6, 10]. Moreover, SA seems to be able to interact with endothelial, B and T cells, leading to the activation of neutrophils and the production of pro-inflammatory cytokines [23]. Age, sex, and ethnicity influence SA colonization. Indeed, significantly higher colonization rates are identified in younger people, in men, and in white populations [6]. Several pathological conditions, such as diabetes mellitus, hemodialytic treatment, end-stage liver disease, obesity, and HIV infection, predispose to nasal SA [6].

To our knowledge, no data on SA colonization in patients with SLE are available so far. Only two studies have been performed in patients with RA. In both analyses, a higher prevalence of SA carriers was found compared to our SLE cohort, and also the prevalence in their control groups was higher than that observed in our HC [9, 11]. Thus, we could reasonably exclude that disease *per se* or differences in the immunosuppressive treatment could justify such an observation. It is more likely that the conditions in which the swab was obtained or differences between the studied populations in age, sex, and ethnicity, could justify such a result.

Intriguingly, we observed that the presence of SA was associated with a specific phenotype of SLE, namely characterized by high frequency of different autoantibodies (anti-dsDNA, anti-Sm, anti-SSA, anti-SSB, and anti-RNP) and by a more frequent renal and skin involvement. Indeed, SA carriers had a significantly higher prevalence of anti-SSA and anti-SSB antibodies, which are known to be associated with cutaneous involvement. In addition, anti-dsDNA and anti-Sm antibodies, which are associated with renal involvement, were present in SA+ patients [24].

It could be hypothesized that SA carriage status induces the production of autoantibodies. No data are available on this topic in patients with SLE. It is possible that SA, by stimulating the type-I IFN pathway, leads to increased production of autoantibodies and therefore to the development of the previously mentioned clinical manifestations [25]. Indeed, dendritic cells (DC), able to recognize pathogens, to activate T cells and to product the type I IFNs, could take up SA through an endocytic mechanism, resulting in the activation of TLR9 signaling. As known, TLR9 is localized at the endosomal level and is involved in autoimmune responses to DNA-associated proteins [22, 26]. Moreover, SA is able, independently of TLR2, to activate human plasmacytoid DC and subsequent IFN-α secretion [27, 28]. The study published by Viau in 2005, evaluating the effects of repeated injection of SA protein A on the (NZBxNZW) F (1) mice lupus model, demonstrated the reduction of anti-DNA IgG production and of proteinuria. The authors suggested that this result could be related to the depression of B-cell response induced by the protein A [29]. As widely demonstrated, SA could interact with both the innate and adaptive immune responses by different virulence factors, among these, the SA protein A, characterized by the presence of immunoglobulin-binding domains, able to bind the Fab of VH3 idiotype antibodies [30, 31].

It should be considered that SA could influence immune response also by the activation of T cells. Data from the literature demonstrated that Staphylococcal enterotoxins (SEs) could bind directly the major histocompatibility complex (MHC) class II of antigen-presenting cells. The presentation to T cells leads to massive non-specific activation of the immune system, by stimulating around 20 % of the naïve T-cell population [32].

The higher prevalence of SA nasal colonization was not associated with any treatment except glucocorticoids. Data from the literature demonstrate that cortisol status can influence susceptibility to infection and that glucocorticoids seem able to reduce the release of pro-inflammatory cytokines, the activation of anti-inflammatory genes, the upregulation of cell adhesion molecules and the downregulation of neutrophil adhesion molecules, thus facilitating the onset of an infective process [33]. Furthermore, Van den Akker and colleagues suggested an association between SA carrier status and polymorphisms of the glucocorticoid receptor gene. Those subjects homozygous for the haplotype 3, which is associated with relative glucocorticoid resistance, had 68 % decreased risk of persistent nasal carriage. Conversely, the genotype combination of the haplotype 5 and the haplotype 1 allele was associated with 80 % increased risk of persistent nasal carriage [34]. It would also be of interest to assess the genotype of the glucocorticoid receptor gene in a population of patients with SLE.

Moreover, it should be considered that glucocorticoid treatment could determine skin abnormalities. In particular, permeability barrier homeostasis and stratum corneum integrity and cohesion could be modified by glucocorticoid treatment, also when performed for a brief period. This could be related to inhibition of the synthesis of epidermal lipid exerted by glucocorticoids [35].

We could not find any significant difference in disease activity nor in the number of flares between SA+ and SA- patients with SLE and only a trend towards higher frequency of persistently active disease was identified in SA+ patients. This result could be related to the single SA assessment performed in the study. SA colonization can vary during the time and it would be of interest to link SA colonization with the occurrence of disease flares. On the other hand, the identification of persistent carriers should be evaluated in relation to the development of more severe chronic damage. In this view, we evaluated disease activity modifications after treatment. The lack of a significant improvement in the disease course evaluated 12 months after the successful eradication with muropicin could be due to the follow up being too short, or to the weak influence of nasal SA on disease activity. Finally, we evaluated only the anterior nares, despite possible colonization in different body sites. However, the primary reservoir for SA in humans is the anterior nares, probably due to the high affinity for nasal epithelial cells. Moreover, nasal secretions also seem to improve the bacterium adherence, in particular, thanks to clumping factor B and iron-regulated surface determinant A. Therefore, in this study we decided to evaluate the colonization of SA exclusively in the anterior nares [36].

A limitation of the present study is the SA identification by a classical morphological evaluation, without molecular characterization. Moreover, the cross-sectional design and the evaluation of nasal SA at a single time point did not allow the exclusion of transient carriers.

## Conclusions

In conclusion, although the small size of the SLE cohort evaluated does not allow definitive conclusions, SA colonization is a relatively frequent event in the course of SLE. The presence of SA seems associated with a peculiar SLE phenotype characterized by cutaneous and renal manifestations. It is not possible to determine whether this is an epiphenomenon rather than a causal factor. Certainly, the skin microbiome deserves deeper investigation, as it may influence disease onset and features.
